# Hedonic Taste in *Drosophila* Revealed by Olfactory Receptors Expressed in Taste Neurons

**DOI:** 10.1371/journal.pone.0002610

**Published:** 2008-07-09

**Authors:** Makoto Hiroi, Teiichi Tanimura, Frédéric Marion-Poll

**Affiliations:** 1 UMR n°1272, Physiologie de l'Insecte: Signalisation and Communication, INRA / UPMC / AgroParisTech, Route de Saint Cyr, Versailles, France; 2 Department of Biology, Graduate School of Sciences, Kyushu University, Ropponmatsu, Fukuoka, Japan; Centre de Recherches su la Cognition Animale - Centre National de la Recherche Scientifique and Université Paul Sabatier, France

## Abstract

Taste and olfaction are each tuned to a unique set of chemicals in the outside world, and their corresponding sensory spaces are mapped in different areas in the brain. This dichotomy matches categories of receptors detecting molecules either in the gaseous or in the liquid phase in terrestrial animals. However, in *Drosophila* olfactory and gustatory neurons express receptors which belong to the same family of 7-transmembrane domain proteins. Striking overlaps exist in their sequence structure and in their expression pattern, suggesting that there might be some functional commonalities between them. In this work, we tested the assumption that *Drosophila* olfactory receptor proteins are compatible with taste neurons by ectopically expressing an olfactory receptor (OR22a and OR83b) for which ligands are known. Using electrophysiological recordings, we show that the transformed taste neurons are excited by odor ligands as by their cognate tastants. The wiring of these neurons to the brain seems unchanged and no additional connections to the antennal lobe were detected. The odor ligands detected by the olfactory receptor acquire a new hedonic value, inducing appetitive or aversive behaviors depending on the categories of taste neurons in which they are expressed *i.e.* sugar- or bitter-sensing cells expressing either *Gr5a* or *Gr66a* receptors. Taste neurons expressing ectopic olfactory receptors can sense odors at close range either in the aerial phase or by contact, in a lipophilic phase. The responses of the transformed taste neurons to the odorant are similar to those obtained with tastants. The hedonic value attributed to tastants is directly linked to the taste neurons in which their receptors are expressed.

## Introduction

While we can distinguish over thousand or more distinctive odors, we perceive tastants as belonging to only five modalities. This is curious because the chemistry of non-volatile molecules is as diverse as that of volatile molecules. Such a difference in perception is the direct consequence of how chemical molecules are sensed by the sensory neurons and ultimately how this information is mapped into the central nervous system. In vertebrates, each olfactory receptor neuron (ORN) expresses a single olfactory receptor gene and any given odor is encoded across a combination of different ORNs [1]. In taste, each sensory cell is sensitive to one taste modality, according to the combination of taste receptors it expresses: T2Rs receptors for bitterness [2], T1Rs for sweet and umami [3] and PKD2L1 ion channels for sourness [4], [5]. Each of these modalities remains quite separate from the others and these divisions can be followed in the upper sensory centers, up to the gustatory cortex [6]. Consequently, in vertebrates, we find a clear chemotopic mapping for olfaction and a broader mapping with fewer modalities for taste. Furthermore, while olfaction (including the vomeronasal organ) is dedicated to detect volatile and mostly lipophilic molecules, taste is tuned to hydrophilic non-volatile molecules commonly found in the food.

Surprisingly, although mammalian and insect olfactory and taste receptors share no sequence similarities [7], their olfactory and taste systems follow the same organization principles [8]. As in vertebrates, most ORN express only one olfactory receptor gene (OR) [9] and ORNs that express the same receptor gene converge onto the same glomeruli, allowing a combinatorial coding up to the higher brain centers [10], [11]. Each gustatory receptor neuron (GRN) encodes broad taste categories, at least phagostimulatory and aversive [12], [13], and co-expresses several gustatory receptors (GRs) [14]. In *Drosophila*, two separate populations of GRNs encode aversive and appetitive information: aversive chemicals are detected by GRNs expressing the GR66a receptor (hereafter called *Gr66a*-GRNs) [15]–[18], while sugars are encoded by GRNs expressing GR5a (*Gr5a*-GRNs) [16], [17], [19]. These populations of neurons project into two distinct brain areas, at least as concerns those located on the proboscis which target the suboesophageal ganglion [16], [17].

In addition to food-related chemicals, insects detect a number of lipophilic non-volatile chemicals for which the receptors are still not known, like cuticular pheromones [20], [21], cuticular compounds that carry nest identity in ants [22] or wax chemicals of plants [23]. Probably as a result of their function to detect hydrophobic molecules, taste sensilla express carrier proteins similar to olfactory carrier proteins found in olfactory sensilla [22], [24]–[27]. These proteins presumably help to transport hydrophobic molecules through the hydrophilic medium surrounding the dendrites to the membrane receptors [25], [26], [28].

Interestingly, insect *Or* receptor genes represent a subset of the lineage of *Gr* genes [15], [29], in contrast to vertebrates, where olfactory and taste receptor genes have diverged earlier in time [30], [31]. These observations suggest that insect ORNs and GRNs have common functionalities, although their respective wiring to the central nervous system is different. They also depart from the structure of vertebrate ORs in that they assume an inverted topology into the membranes, their N-terminus being intracellular rather than extracellular [7], [32]. This inverted topology may prevent these receptors to link to G proteins and recent observations made by heterologous expression in different expression systems indicate that they indeed form heteromeric ligand-gated channels [33], [34].

In this work, we asked if *Ors* can be expressed in GRNs and if these GRNs would then acquire the capability of sensing volatile molecules. To this end, we used the *Drosophila* olfactory receptor *Or22a* [11] sensing molecules to which GRNs are naturally blind and expressed it in GRNs detecting either sugar or bitter chemicals. Using an electrophysiological technique to record from insect olfactory sensilla, and neuroanatomical and behavioral approaches, we demonstrate that this olfactory receptor is functional in taste cells and that odorants modify the feeding behavior depending on which taste neurons in which they are expressed. As expected from previous observations [7], [35], the olfactory receptor protein needs to be co-expressed with *Or83b* to be functional. Our electrophysiological observations demonstrate that odorant molecules are detected both by contact and at short distance. Considering the differences in morphology between olfactory and taste sensilla, it is surprising that transformed taste neurons could sense odors. From the work of Benton *et al* [7], we know that ORs can be expressed in GR-expressing neurons. However, these GR receptors (*Gr22a* and *Gr63a*) are involved in sensing CO_2_ in the air on *Drosophila* antennae and their neurons are true olfactory neurons projecting into the antennal lobe. In this experiment, we expressed ORs in sensilla designed to detect chemicals by contact and not in the vapor phase. If it is expected that odorant molecules enter freely into olfactory sensilla to reach the sensory neurons, our observations indicate that odorants can also enter into taste sensilla and reach taste neurons. As a result of this ectopic expression, odorant detected by the odorant receptor acquire a new hedonic value depending on the taste population in which it is expressed.

## Results

### Tungsten electrode recordings from taste sensilla stimulated with odorants

**Figure 1:**
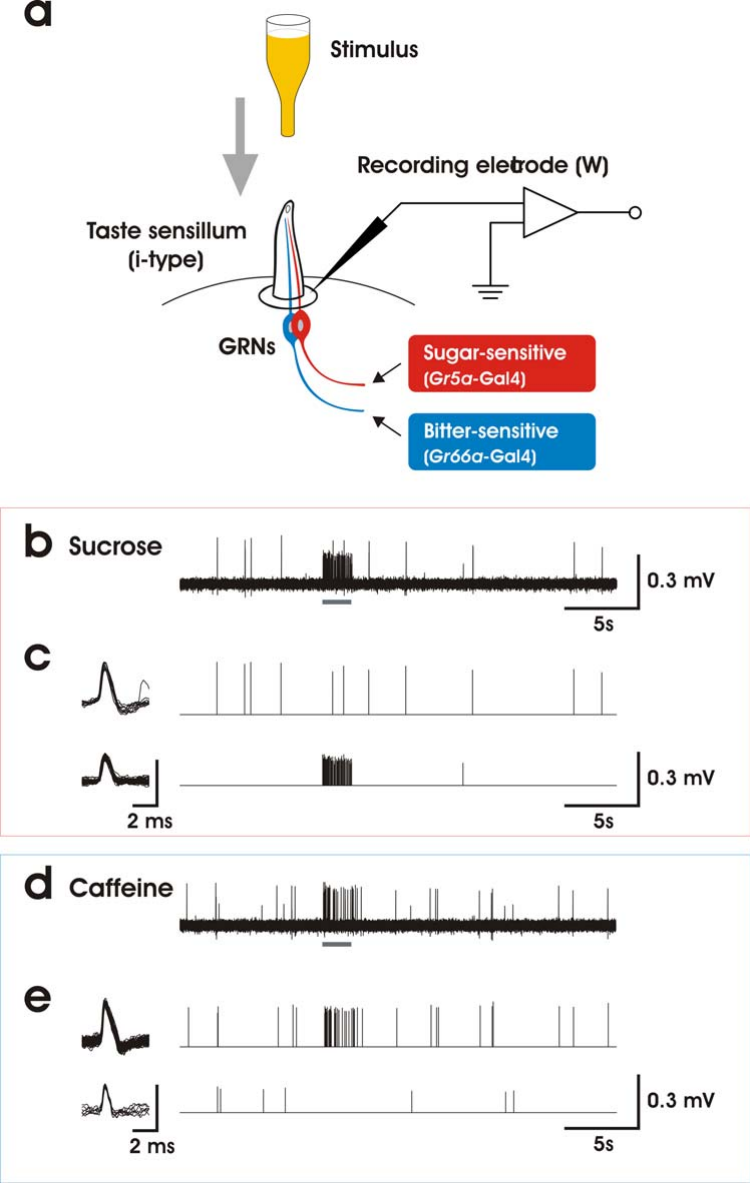
Electrophysiological responses of *Drosophila* i-type taste sensilla to sugar and bitter substances. a. Schematic diagram of the recording setup. Electrical signals were recorded from an electrolytically sharpened tungsten electrode inserted through the cuticle at the base of a taste sensillum. To stimulate the GRNs, the tip of the sensillum was capped during 2–5 s with a glass capillary filled with a stimulating solution. The i-type sensilla house only two GRNs, which elicit spikes of different amplitudes that were separated using custom software routines (23) as shown in 1c and 1e. The sugar-sensing GRN expresses *Gr5a* and the bitter-sensitive GRN expresses *Gr66a* [40]. b. Sample recording with 100 mM sucrose (stimulus = 2 s horizontal grey bar). c. Sucrose elicits a response only in the cell that fires spikes of smaller amplitude (lower trace); superimposed spikes (left column) and time-series extracted (central trace and bars) after software spike separation. d. Sample recording with 1 mM caffeine. e. Caffeine elicits a response only in the cell that produces spikes of larger amplitude. Vertical bars = 0.3 mV.

Recordings from insect taste sensilla are usually performed using the tip-recording method [36], in which the same electrode contains the stimulus and an electrolyte to conduct electrical currents. Since many odorant molecules are not water-soluble, we uncoupled the stimulation and the recording, using a two-electrode configuration: a fine tungsten electrode was inserted through the cuticle at the base of a taste sensilla to record from the nerve cells while another capillary electrode, containing tastant or odorant molecules in solution within a lipophilic solvent, was briefly brought in contact with the tip of the hair to stimulate them (Fig. 1a).

**Figure 2:**
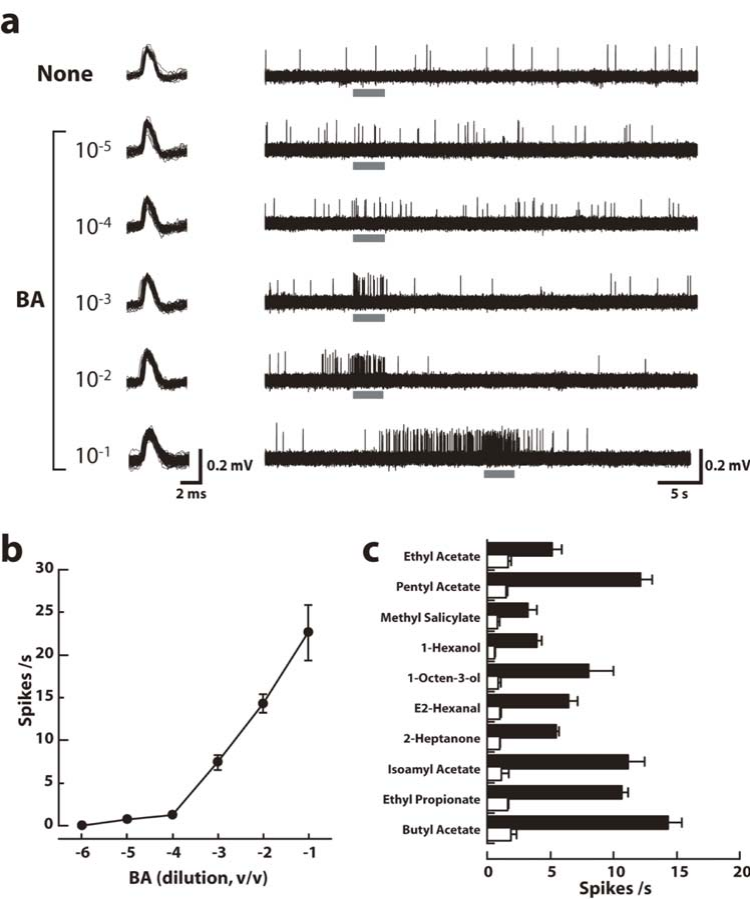
Responses to odors in bitter-sensitive GRNs expressing *Or22a* co-expressed with *Or83b*. a. Sample recordings from an i-type gustatory sensillum expressing *Or22a*+*Or83b* driven by *Gr66a*-Gal4, and stimulated with increasing concentrations of butyl acetate diluted in paraffin oil (dilution of 10^−5^ to 10^−1^, solvent: paraffin oil only). The stimulation is marked by a horizontal grey bar. Note that the response started before the capillary contacted the sensillum at the higher concentrations (10^−2^ and 10^−1^), demonstrating that air-borne chemicals stimulate the altered GRNs at high concentrations. b. Sensitivity of the altered GRNs to butyl acetate (BA) (mean±S.E.M.; n = 4 to 19 trials). Ordinates: firing frequency measured during the 2 s stimulation. Abscissa: BA dilutions along a logarithmic scale. c. Odor response spectrum of GRNs expressing *Or22a*+*Or83b*; all odorants were diluted 10^−2^ in paraffin oil. Black bars: OR22a+OR83b; Open bar: wild type, mean±S.E.M.; n = 4∼17.

We examined the responses of taste sensilla located on the proboscis of adult flies, targeting sensilla which contain only two GRNs in order to obtain unambiguous results concerning the identity of the cells active in our recordings [37]–[39]. These i-type sensilla are located at the periphery of a sensilla field that comprises about 32 hairs on each lobe of the proboscis. In i-type sensilla, one GRN responds to sucrose (small amplitude spikes: Fig. 1b, c) while the second GRN responds to bitter substances [40], like caffeine (larger spikes: Fig. 1d, e). In wild-type flies, none of these taste cells responded to any chemicals chosen from a panel of odorants detected by native olfactory receptor neurons expressing OR22a (*Or22a*-ORN) [11] (Fig. 2c: white bars); they also did not respond to paraffin oil, which served as a solvent (Fig. 2a: “none”).

### Electrophysiological responses of transformed GRNs to odorants

**Figure 3:**
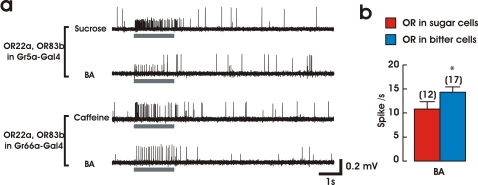
Responses to odors in taste neurons expressing *Ors* under different Gal4 drivers. a. Taste neurons expressing *Or22a*+*Or83b* in sugar-sensing neurons (driven by *Gr5a*-Gal4) respond to sugar and to butyl acetate (first two traces: smaller amplitude spikes); taste neurons expressing *Or22a*+*Or83b* in bitter-sensing neurons (driven by *Gr66a*-Gal4) respond to caffeine and to butyl acetate (lower two traces: larger amplitude spikes). Gray bar = 2 s stimulus. Horizontal black bar: 1 s; vertical bar: 0.2 mV. b. Comparison of the responses of altered GRNs to butyl acetate depending on the driver, *Gr5a*-Gal4 (“sugar cells”) or *Gr66a*-Gal4 (“bitter cells”) (mean±S.E.M.; n = 12, 17 trials). There is a small difference in response intensity to butyl acetate in *Gr5a*- and *Gr66a*-GRNs (*: P = 0.039, Student's t-test).

We used the Gal4/UAS system to ectopically express ORs in a particular set of gustatory receptor cells and then tested if transformed GRNs responded to butyl acetate, one of the ligands detected by *Or22a*-ORNs. In flies expressing *Or22a*/*Or83b* in *Gr66a*-GRNs, the caffeine-sensitive neurons responded to butyl acetate in a dose-dependent manner (Fig. 2a, b). The firing activity reverted quickly to the background level as soon as the contact with the stimulus was broken except at the lowest dilution (1∶10). When *Or22a* or *Or83b* were expressed separately, no response to butyl acetate was observed (data not shown). These transformed GRNs retained their capacity to respond to sugars or to bitter compounds (see sample recordings in Fig. 3a). Thus, an odorant can activate GRNs expressing OR22a/OR83b, while these GRNs retain their innate sensitivity to contact chemicals. Here, butyl acetate was detected as a stimulus upon contact with the tip of the capillary tube containing the odor ligand in solution within paraffin oil, except at higher doses (10^−2^ and 10^−1^ dilution) where molecules in the vapor phase could be detected before the contact occurred (Fig. 2a). In all subsequent experiments, we avoided vapor stimulation by directing a constant flow of humidified air onto the preparation.

OR-expressing GRNs also responded to the other odorants [11], [41] known to elicit a response in *Or22a*-ORNs (Fig. 2c). The response profile of these GRNs appear to be qualitatively similar to that of *Or22a*-ORNs [see 11: Fig. 1c]. Thus, OR22a associated with OR83b [7], [35] behaves as a functional receptor sensing odors.

We then examined if GRNs that sense sugars could be transformed in the same way. We used a *Gr5a*-Gal4 strain to express *Or22a* and *Or83b* in sugar-sensitive GRNs [42], [43]. In flies expressing both *Or22a* and *Or83b* driven by *Gr5a*-Gal4, the spikes elicited during stimulation with butyl acetate are of the same amplitude as those elicited by sugars (Fig. 3a, upper two traces). In *Gr66a*-Gal4 driven GRNs expressing *Or22a* and *Or83b*, generated spikes identical to ones elicited by bitter compounds (Fig. 3a, lower two traces). This confirms that one odorant (butyl acetate) could excite different set of GRNs depending on which GRN expresses *Or22a*. The odor-evoked responses from *Gr66a*-GRNs seem slightly higher than ones from *Gr5a*-GRNs (Fig. 3b). This difference could be due to different expression levels of GAL4.

### Projections from transformed-GRNs to the brain

**Figure 4:**
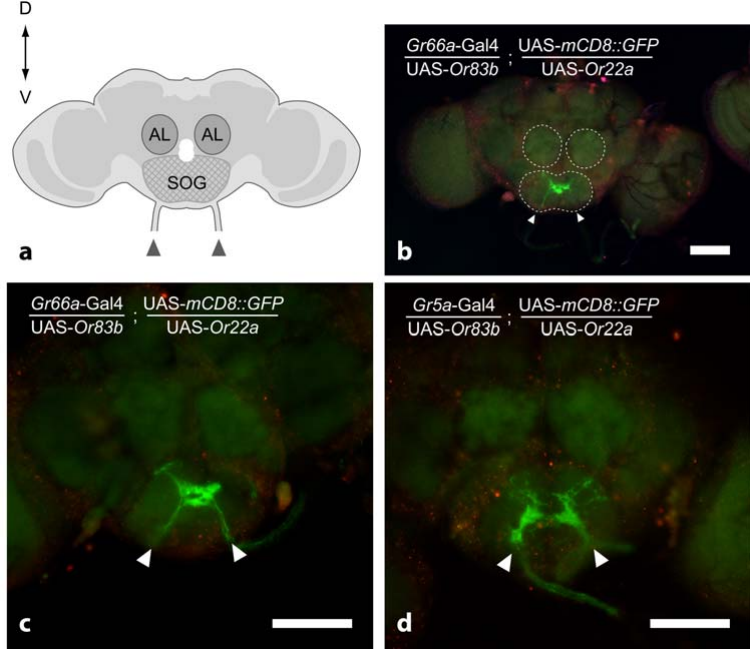
Ectopic expression of *Or22a* and *Or83b* in GRNs does not alter the projection pattern of the GRNs. a. Schematic diagram of the frontal view of a fly brain. Dorsal is to the top. Arrow heads indicate incoming fibers of GRNs in the proboscis. AL = antennal lobe, SOG = suboesophageal ganglion. b. Whole-mount of a fly brain, showing projection of *Gr66a*-positive GRNs that co-express *Or22a* and *Or83b* in the SOG. *Gr66a*-GRNs were labeled by mCD8::GFP. Areas surrounded by dotted line are ALs and SOG. This figure and the followings are single pictures from the microscope. The red signal is used here to obtain the outlines of the brain. c. Close-up view of the SOG and ALs in b. d. Projection of *Gr5a*-GRNs expressing *Or22a* and *Or83b* in the SOG. Both type of GRNs (*Gr66a* and *Gr5a*) were not affected in their projection patterns by co-expressing the Ors in their projection patterns. Scale bars in b, c and d = 50 µm.

In order to test if transformed GRNs would project to the central nervous system (CNS) as normal taste neurons or as odorant receptor neurons (ORNs), we observed two further *Drosophila* lines, bearing a membrane-targeted mCD8-GFP [44] driven by *Gr5a*- or *Gr66a*-Gal4, as well as the two odorant genes, *Or22a* and *Or83b*. We used a confocal microscope to identify their targets in the brain. No projections were found within the antennal lobes, either in the DM2 glomerulus which receives projections from Or22a-expressing olfactory neurons [45] or in the other glomeruli (Fig. 4b). Intense markings were found in the suboesophageal ganglion, both in *Gr5a*- and in *Gr66a*-Gal4 driven flies. The neurons labeled by GFP projected respectively in the central omega-shaped area previously described for *Gr66a*-GRNs (Fig. 4c) and lateral area (Fig. 4d) described for *Gr5a*-GRNs [17]. This confirms that the modified taste neurons not only conserved their physiological responses to their natural ligands but also retained the mapping described earlier for *Gr66a*- and *Gr5a*-GRNs [17].

### Feeding behavior of flies with transformed-GRNs

Electrophysiological and neuroanatomical data suggest that flies expressing the *Ors* in *Gr66a*-GRNs recognize butyl acetate as a bitter stimulus while those with altered *Gr5a*-GRNs perceive it as a sweet stimulus. This hypothesis was tested by monitoring feeding preferences driven by odorants and/or by normal tastants in wild or transformed flies. When hungry flies are placed in a Petri dish with two disks of agar containing sugar, they spend more time around the agar disks than elsewhere in the arena. By adding odorants to one of the food disks, we could then monitor if their behavior was modified by computing the ratio of the time spent during the experiment around these disks. In order to minimize interferences with antenna-based odor preferences, the flies used in these experiments were surgically deprived of their antennae. These flies were not deprived of their palps, which bear ORNs for which butyl acetate is a minor stimulant [46].

**Figure 5:**
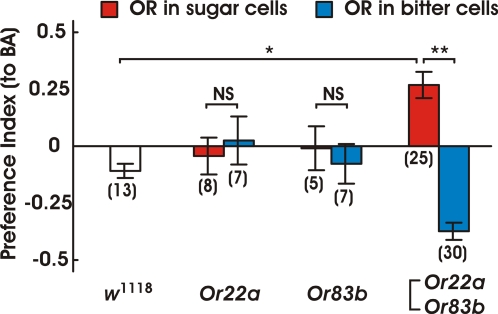
Behavioral choices expressed by flies between a control agar disk and disk treated with butyl acetate. Ordinates: ratio of the density of presence of flies on odorant-treated or non-treated food (mean±S.E.M.; number of trails for each is noted in the figure). Abscissa: Wild-type (*w* ^1118^) and flies expressing *Or22a*, *Or83b* or *Or22a*+*Or83b* under the control of *Gr5a*- or *Gr66a*-Gal4. Flies expressing both *Or22a* and *Or83b* showed an altered preference to butyl acetate as compared to the wild-type (*w* ^1118^). Depending on the GRNs that express the *Ors*, flies exhibited the opposite preferences to the same odorant. BA = Butyl acetate. * P<0.05, ** P<0.01 with the Tukey-Kramer multiple comparisons test.

In these conditions, we observed that flies expressing *Or22a*+*Or83b* significantly changed their feeding behavior (Fig. 5). When both *Ors* were expressed in sugar-sensitive GRNs (*Gr5a*-Gal4), flies aggregated around the food treated with the odorant. Likewise, when these *Ors* were expressed in bitter-sensitive GRNs (*Gr66a*-Gal4), flies approached the food and moved away after having examined it, aggregating around the non-treated food disk. Flies expressing a single *Or* (*Or22a* or *Or83b*) did not behave differently from wild-type flies (*w^1118^*).

In summary, odorants sensed by the ectopically-expressed *Ors* induce behaviors similar to those elicited by sugars or by bitter substances, depending on the identity of the GRNs in which they are expressed. These modified GRNs are fully functional as regards to taste sensing. OR expression did not affect axonal projection of either *Gr5a*- or *Gr66a*-GRNs (Fig. 4).

## Discussion

Our experiment provides the first direct evidence that olfactory receptors are functional in true taste neurons of *Drosophila*. These neurons respond to odorants dissolved in paraffin oil upon contact, as if odorants were sapid molecules, and they can even respond to these molecules in air at close range. Our observations indicate that the hedonic value that was associated with the detection of the odor is changed according to the identity of the GRNs expressing this receptor.

Our results are consistent with and extend previous results published by Benton *et al.* [7]. Benton *et al.* expressed olfactory receptors in several classes of antennal neurons, including mechanosensory neurons of the Johnston organ and CO_2_-sensing neurons. Olfactory receptors like *Or22a* or *Or43a* need to be co-expressed with *Or83b* to be correctly addressed to the dendritic membranes and to induce functional responses to the proper odorant ligands. Benton *et al.* expressed the olfactory receptor *Or43a* (with *Or83b*) in antennal neurons expressing *Gr21a*; these neurons respond to CO_2_ in the air and acquire the property of responding to cyclohexanol which is a ligand for *Or43a*. Although *Gr21a* and its partner *Gr63a* [47] are classified as a taste receptors, these neurons should be considered as olfactory: (i) they are housed into sensilla ab1C [41] which are lacking a terminal pore considered as characteristic to taste sensilla [48] and (ii) they project into the antennal lobe to the DM2-glomerulus while antennal taste sensilla in other insects project into the suboesophageal ganglion [49]–[51]. Nonetheless, these CO_2_-sensing sensilla express “gustatory” receptors which are functional in the absence of *Or83b* [47]. While Benton *et al.* demonstrated that ectopic olfactory receptors are functional by population measurement using calcium imaging on the antennal lobe, we used single-sensillum recordings that gives a greater temporal resolution. Lastly, our work extend Benton *et al.*'s work, by analyzing how the hedonic value of the odorants is changed after miss-expressing ORs into GRNs.

One important aspect of these experiments is that altered GRNs transduce odorants despite the obvious structural differences between olfactory and taste sensilla *e.g.* a single terminal pore for taste sensilla *vs.* a host of minute pores on the hair shaft for olfactory sensilla [7], [52]. The fact that volatile molecules can enter the terminal pore and stimulate taste neurons has received scant attention, except for reports showing that plant odors stimulate taste receptor neurons of tobacco hornworm larvae, *Manduca sexta* [53], the Colorado potato beetle, *Leptinotarsa decemlineata* (Say) [54] and the blowfly [28]. Further indications that taste sensilla may sense lipophilic molecules and odorants come from molecular studies that repeatedly report the presence of odorant-binding proteins in various taste sensilla of insects [24]–[26], [28], which contribute to the transfer of chemicals from air to the sensillum lymph [22]. While the tip-recording technique requires the use of lipophilic solvents [22], [28] that may damage the distal membrane of the taste cells, the technique we used here should be suitable to record the responses of GRNs to other lipophilic compounds like cuticular pheromones [21] or water-insoluble compounds from plants.

OR83b is an essential partner to OR22a and other odorant receptor proteins [35], [55]. Benton *et al.* [7] have shown these molecules form a dimer and adopt *in vivo*, a topology where their N-termini and most conserved loops are in the cytoplasm; this observation was confirmed by another approach [32]. This conformation suggested that signaling downstream of the ORs was non-canonical, a prediction that has been recently confirmed by two independent studies using *in vitro* heterologous expression systems [33], [34]. That OR receptors can induce spiking activities in taste neurons is therefore not surprising: these dimers form channels that when gated by an odorant, may generate current sufficient to induce a receptor potential and trigger the firing of action potentials. However, evidence is still missing about how these ORs are activated *in vivo*, especially considering that in addition to the odorant-gated channel activation [33], [34], ORs may interact with more classical transduction pathways like cAMP or cGMP [34], or even phospholipid signaling [56]. From this perspective, *Drosophila* taste neurons represent a useful expression system to evaluate the specificity of olfactory receptors, as it provides cells fully equipped with compatible transduction pathways whose activities can be monitored by extracellular recording techniques or possibly by patch-clamp as done in fleshfly sugar-sensing GRNs [57].

Flies expressing olfactory receptors within subsets of taste neurons sharing the expression of the same GR should be particularly useful for understanding how the taste modalities are encoded at the periphery. Although the functional separation between sugar-sensing and bitter-sensing seems quite natural, it rests on chemical characteristics that may overlap. For example, NaCl was found to stimulate sugar-sensing cells at low concentration and bitter-sensing cells at high concentrations [40]. Likewise, a number of artificial sweeteners are stimulating both sugar-sensing cells and bitter sensing-cells in humans and in flies [58]. Because several *Gr* are co-expressed in *Gr66a*-GRNs and in *Gr5a*-GRNs [42], [59], [60], it is likely that more than one neuron detects the same molecule within a sensillum. The use of a heterologous receptor as a reporter gene for a given *Gr* has the advantage of activating only one cell without the confounding activity of the other cells [61].

While previous observations showed that impairing the expression of *Gr5a* or *Gr66a* in taste neurons changed the behavioral responses to sugars or to bitter substances and as well as the activities of the neurons projecting in the brain after “ensemble” stimulations [16], [17], our experiments directly demonstrate that individual GRNs which express *Gr5a* and *Gr66a* are different and respond to sugar and to bitter compounds. Our study, as well as other studies [12], [16], [17], [61], [62], indicates that taste sensory cells of insects encode broad qualities similar to those found in vertebrates [2], [63].

If the hedonic value of tastants is hard-wired in insects, it would be interesting to know how fast insects can adapt to substances that are detected within the wrong category. This is a critical question if one wishes to use bitter substances for protection against pest insects. A number of observations have established that phytophagous insects can adapt to bitter substances if they are not toxic, for example by reducing the sensitivity of their taste neurons [64] or by increasing the response to a feeding stimulant specific to their host plant based on their experience [65]. More intriguing are situations where insects become repelled by appetitive stimuli. For example, cockroach strains resistant to a bait associated with an insecticide were found to become repelled by glucose [66]. Lastly, the hedonic value of a given stimulus might also be context-dependent as shown by recent observations of flies preferring to lay eggs in a medium containing a bitter substance over a medium containing sucrose [67]. Experience-dependent changes in the sensitivity of individual taste neurons, genetic changes affecting the expression of taste receptors and short-term memory might be three major driving mechanisms that allow insects to cope with this hard-wired system and to adapt to their environment.

## Materials and Methods

### Fly strains

*Drosophila melanogaster* stocks were reared on standard cornmeal-agar-glucose medium at 23°C. *w^1118^* flies were used as the control strain. For the ectopic expression of *Or22a* and/or *Or83b* in GRNs, flies which carry both UAS-*Or83b* (supplied by Leslie B. Vosshall) and UAS-*Or22a* (supplied by John R. Carlson), balanced with CyO and TM6B, respectively, were crossed with either *Gr5a*-Gal4 or *Gr66a*-Gal4 flies. To visualize the projection patterns of GRNs expressing Ors, *Gr5a*- or *Gr66a*-driven GRNs were labeled by crossing in UAS-mCD8::GFP. We used the following genotypes for electrophysiology: *w^1118^*; *+* ; *+* (Fig. 1b–e: control flies), *w^1118^*; *Gr66a*-Gal4/UAS-*Or83b*; UAS-*Or22a*/(TM6B or MKRS) (Fig. 2 & 3a: *Gr5a* - *Or22a*+*Or83b* flies), *w^1118^*; *Gr5a*-Gal4/UAS-*Or83b*; UAS-*Or22a*/(TM6B or MKRS) (Figure 3a: *Gr5a* - *Or22a+Or83b* flies). For brain imaging, we used *w^1118^*; *Gr5a*-Gal4/UAS-*Or83b*; UAS-*Or22a*/UAS-mCD8::GFP and *w^1118^*; *Gr66a*-Gal4/UAS-*Or83b*; UAS-*Or22a*/UAS-mCD8::GFP. For the behavior assay, we used *w^1118^*; *+*; *+* as control, *w^1118^*; *GrX*-Gal4/*CyO*; UAS-*Or22a*/(TM6B or MKRS) as *Or22a* flies, *w^1118^*; *GrX*-Gal4/UAS-*Or83b*; TM6B/MKRS as *Or83b* flies and *w^1118^*; *GrX*-Gal4/UAS-*Or83b*; UAS-*Or22a*/(TM6B or MKRS) as [Or22a+Or83b] flies, where *GrX* is *Gr5a* for sugar cells and *Gr66a* for bitter cells.

### Chemicals

All odorants and chemicals were obtained from Sigma Aldrich. Hydrophilic compounds were dissolved in water. Lipophilic compounds were diluted in paraffin oil.

### Electrophysiological recordings

Recordings were made from i-type sensilla on the labellum. Adult flies were anesthetized by ice and the severed head was impaled on a reference electrode. The proboscis was exposed and maintained in place between a glass plate and a rod mounted on a micromanipulator, and oriented under a stereomicroscope (Leica M10, ×250, Germany). In order to record the responses of GRNs from odors diluted in paraffin oil, we inserted an electrolytically sharpened tungsten electrode at the base of taste sensilla, connected to a custom-built preamplifier and further amplified (×1000) by a CyberAmp 320 amplifier (Axon Instruments, USA) using 8 dB Bessel band-pass filters from 0.1 Hz to 2800 Hz. The timing of the stimulation was controlled by a stepper motor (PC-5N, Narishige, Japan) that advanced and retracted the stimulus capillary under the control of the computer. Except for data presented in Fig. 2a, a gentle flow of humidified air was directed on the preparation in order to prevent stimulation of the GRNs before a contact was made between the capillary and the hair. Electrical signals were sampled at 10 kHz on a computer (DT9803 USB A/D card, Data Translation, USA) and analyzed using custom-built software to detect and sort spikes. The response of the neurons was assessed by counting the number of spikes elicited during the stimulation. The results were first checked for their normal distribution by the F-test and then compared using Student's t-tests.

### Neuroanatomical projections from transformed neurons

Dissected whole-mount labella and brains were fixed in 4% formaldehyde in PBS at room temperature for 3 h. The tissues were washed in PBS and mounted in 60% glycerol. GFP images were captured by using an LSM 510 laser confocal microscope (Zeiss, Germany). Fluorescence was emitted by 488 nm and observed with a bandpass filter at 505–550 nm (green) and a long pass filter at 650 nm (red). The green channel indicates fluorescence emitted by GFP while the red signal helps to recognize the outline of the brains by autofluorescence.

### Behavioral tests

Two disks of agar (10 mm dia., 1–2 mm thickness) cut from a layer of 1% agar containing 30 mM sucrose were placed in an experimental arena (80 mm dia. glass Petri dish). One of the disks received 50 µl of butyl acetate diluted at 10^−2^ in hexane, while the other disk received the same amount of hexane. In each experimental arena, we placed 20–40 adult flies, deprived of food for 20–24 hrs. The arena was monitored by a digital camera (Logitech Inc., USA) sampling one image (240×320 pixels) per 2 s for 20 min and storing the images on a disk. The files were then analyzed using a custom program running under Matlab 6.5 (The Mathworks, Inc., USA) that isolated the flies from the background and then converted the results into binary images. This allowed us to monitor areas where the flies spent the most time by simply counting black pixels within two “regions of interest” around the food disks. The distribution of this density of presence was compared using a simple index: SUM [(D area A) − SUM (D area B)] / [SUM (D area A+D area B)], where D = total number of pixels set to 1 in the corresponding area. These data were compared statistically to the control by means of the Tukey-Kramer method which accepts comparisons among groups which are of unequal sample size.
